# Therapeutic Futility in Terminal Cancer Patients: A Retrospective and Observational Study

**DOI:** 10.7759/cureus.14073

**Published:** 2021-03-24

**Authors:** Joana Graça, Leonor Vasconcelos de Matos, Ana Mafalda Baleiras, Filipa Ferreira, Rui Costa, Marta M Pinto, Ana Martins

**Affiliations:** 1 Medical Oncology, Centro Hospitalar Lisboa Ocidental – Hospital São Francisco Xavier, Lisbon, PRT; 2 Internal Medicine, Hospital da Luz, Lisboa, PRT

**Keywords:** advanced cancer, hospice and palliative care, supportive and palliative care, end of life and hospice care, therapeutic interventions

## Abstract

Introduction

Advanced cancer patients often need therapy for symptomatic control, in addition to cancer and other disease treatments. As the cancer disease progresses and life expectancy decreases, there should be a change in the goal of care. If this change is not accompanied by therapeutic adjustments, there is a risk of maintaining useless and ineffective treatments, as well as potential harmful drug interactions. This study analyzed the prevalence of therapeutic futility in patients with advanced cancer disease.

Materials and methods

This was a retrospective and observational single-center study, that included advanced cancer patients who died during the hospital stay, at a University Hospital in Lisbon, Portugal. Demographic and clinical data were collected. A Palliative Prognostic Score (PaP) was used to stratify patients according to their prognosis group. An analysis of the prescribed therapy was performed to quantify the “potentially inappropriate medications” (PIMs) and “inappropriate medications” (IMs), at admission and 24 hours prior to the patient’s death.

Results

Over 140 patients were included. On the first day of hospitalization, 119 patients (85%) were exposed to at least one IM or PIM and 100 patients (71%) were still exposed to at least one IM or PIM in the last 24 hours of life. Regarding chemotherapy, 66 patients (47%) had treatment in the last two months of life, 38 (27%) in the last month, and 17 (12%) in the last two weeks prior to death.

Therapeutic simplification (suspension of IMs and reduction of at least 50% of PIMs during hospitalization) was performed in 43% of the overall population and was higher in PaP score group C, but not statistically significant (p=0.09). The patient's inclusion in PaP score group C and inpatient consultation by the palliative care team were independent predictors of therapeutic simplification.

Discussion

There is an effort to achieve greater therapeutic suitability in palliative patients. However, many patients maintain futile and disproportionate therapy at the end of life (EoL). In many cases, systemic cancer treatment is performed until quite late in the course of the disease. The prescription of PIMs was significantly higher than that of IMs, which could be expected given their definition. A shorter life expectancy at admission led to a greater therapeutic simplification, as well as an intervention by the Palliative Care Team, which can be explained by the more focused approach towards quality-of-life improvement and symptomatic control. Different than expected the prescription of supportive therapies at hospital admission was not a predictor of therapeutic simplification. Although there was a reduction in IMs and PIMs in the studied population, and therapeutic simplification occurred in one fraction of the patients, the fact is that more than half of the patients evaluated did not undergo therapeutic simplification as defined in this work.

Conclusion

It appears that there is an effort to achieve greater therapeutic suitability in palliative patients, however, many patients maintain futile therapy at the EoL. It is of paramount importance to change the standard of care in this setting, to privilege a more patient-focused approach and tailored therapy, and to prioritize symptomatic control and quality-of-life improvement.

## Introduction

Polypharmacy is a real and growing problem. It affects mainly the elderly, which often have multiple medical conditions, with the need to carry out specific therapies to treat each one of them and to prevent medium- and long-term consequences. This reality also applies to cancer patients.

Patients with advanced cancer disease often need therapy for symptomatic control, in addition to cancer and other disease treatments. As the cancer disease progresses and life expectancy decreases, there should be a change in the goal of care. If this change is not accompanied by therapeutic adjustments, there is a risk of maintaining useless and ineffective treatments, as well as potential harmful drug interactions [1,2].

When the goal of treatment is symptomatic control and improvement in functionality and quality of life, the maintenance of drugs meant for the prevention of long-term complications from other comorbidities no longer makes sense. In addition, there are pharmacokinetic and pharmacodynamic changes, mainly due to a reduction in glomerular filtration rate, that must be considered in the end-of-life (EoL) period, since it may increase the risk of toxicity [3].

According to the literature, a futile medical intervention is defined as an “intervention that no longer provides a clear benefit for the patient, does not reach its purpose, and has the potential to be harmful” [4]. Therapeutic simplification can be defined as a process of therapeutic adjustment, of “deprescribing,” which means the withdrawal of potentially inappropriate or unnecessary drugs, that have a negative balance of benefits and harms, minimizing adverse effects and harmful drug interactions [5,6].

Assessing the patient's prognosis can be useful to identify the need to perform therapeutic analysis of ongoing therapy and, eventually, therapeutic simplification. Over the past decade, several prognostic scores have been presented and validated, namely the Palliative Prognostic Score (PaP score) [7], the Palliative Prognostic Index (PPI) [8], and the Palliative Performance Scale (PPS) [9].

A multicenter European study of 2282 cancer patients reviewed the drug prescription in patients with severe chronic pain, treated with strong opioids. They found that these patients had an average of 7.8 drugs (from one to 20) and that more than a quarter had 10 or more drugs. The most commonly co-administered drugs were proton pump inhibitors, laxatives, corticosteroids, paracetamol, non-steroidal anti-inflammatory drugs, prokinetic agents, benzodiazepines (BZD), anticoagulants, antibiotics, antiepileptics, diuretics, and antidepressants. Approximately 45% of patients received unnecessary or potentially unnecessary drugs, and about 7% received duplicate drugs or antagonists [10]. 

Another retrospective study assessed the hospitalized cancer patients with advanced disease, at the EoL, who were under polypharmacy. In the nine days before the patient's death, 95% of them had 11 different drugs prescribed daily. Although this number dropped considerably on the last day of life, 61% of patients were still medicated with four or more drugs [11].

The hypothesis that led to this investigation was that cancer patients at the EoL may not have a therapeutic plan suitable to their needs, being treated with multiple drugs and with unnecessary therapy. This study intended to analyze the therapeutic futility in patients with advanced cancer disease. The main goal was to determine if there was a therapeutic simplification in EoL cancer patients and if there are predictors of therapeutic simplification in this setting.

## Materials and methods

Study population

Patients over 18 years of age were included, with a diagnosis of advanced cancer (metastatic solid tumor), and who had died during the hospital stay, at the Medical Oncology Unit of a University Hospital (São Francisco Xavier), in Lisbon, Portugal, between January 2015 and September 2020.

Study design

This was a retrospective and observational single-center cohort study. Demographic and clinical data were collected, as well as causes of hospitalization and significant complications, infectious or other. The analysis of the prescribed drugs and the quantification of inappropriate or potentially inappropriate medications (PIMs) was made through the evaluation of the collected data. This assessment was made at admission and 24 hours prior to the patient’s death.

According to the existing data in the literature, drugs were classified as either inappropriate or potentially inappropriate. “Inappropriate medications” (IMs) when the treatment did not have a beneficial effect on symptom control, quality of life, or survival; these included statins, anti-diabetic medication, hormone replacement therapy, vitamins, and some minerals (except potassium, calcium, magnesium, and iron) [10-12].

“Potentially inappropriate medications” those whose futility cannot be definitively attributed retrospectively, but which is likely if there is a poor performance status (PS) (Karnofsky Performance Status (KPS) <50%) and/or reduced life expectancy. Cancer treatment, megestrol acetate, cardiovascular drugs, anticoagulants, gastric protectors (H1 blockers, proton pump inhibitors, antacids), and allopurinol were considered PIMs. The cardiovascular drugs included were anti-hypertensive agents (except diuretics), antiarrhythmic drugs, and antiaggregants [10-12].

The drugs used for symptomatic control were also explored and were categorized into the following pharmacological groups: opioids, non-opioid analgesics, antidepressants, antiepileptic drugs, antipsychotics, sedatives, BZD, laxatives, corticosteroids, and antiemetics. It was also analyzed whether there was an intervention by the Palliative Care Team. It was considered an intervention if there was an inpatient consultation to assess symptomatic control, with or without therapeutic adjustment.

To estimate the prognosis of the patients, the PaP score was used. This score was chosen because of the easier application considering the data available in the clinical records. This score utilizes six criteria (dyspnea, anorexia, KPS, Clinical Prediction of Survival (CPS) in weeks, total white blood cell (WBC) count, and lymphocyte percentage), to create a numerical score from 0 to 17.5 (higher scores predicting shorter survival). The patients were divided into three groups, based on survival prediction, in group A (0 - 5.5), B (5.6 - 11), and C (11.1 - 17.5), corresponding to a 30-day survival probability of >70%, 30-70% and < 30%, respectively [13].

The score was applied using the medical information recorded at hospital admission, retrospectively, and the CPS, despite being a subjective parameter, was extrapolated from the data. If the possibility of starting or changing cancer treatment was described, it was presumed a CPS of 12 weeks; if it was described that the patient did not have a suitable clinical condition for cancer treatment, it was assumed a CPS of less than 12 weeks; if there was a description of "end-of-life patient," "terminal patient," "goal of care: comfort,” a CPS of less than four weeks was established; if "patient in the last days or hours of life" was described, a CPS of less than two weeks was assumed.

The patients were stratified according to their prognosis group, to allow a separate analysis, especially of the group with the worst prognosis at admission (PaP score group C). Therapeutic simplification was defined as the suspension of all IMs and reduction of at least 50% of PIMs during hospitalization.

Statistical analysis

Analysis of normality was undertaken using the Shapiro-Wilk test. To compare the study groups, Wilcoxon rank-sum test was used for continuous variables and the χ^2^ test of independence for categorical variables. Logistic regression models were performed, to study the effect of explanatory variables on therapeutic simplification, with a stepwise approach for multivariate analysis. For each factor, we have calculated the adjusted odds ratio (OR) and 95% confidence interval (CI) using maximum likelihood estimation. All analysis was performed using Stata 15.1 software (StataCorp LLC, College Station, TX). All results with a p-value less than 0.05 were considered statistically significant.

## Results

We included 140 patients. Patient demographic and clinical characteristics are shown in Table 1. 

**Table 1:** Demographic and clinical characteristics of the overall population and of the PaP Score Group C population. PaP: palliative prognostic score.

Characteristics	Overall Population (n=140)	PaP Score Group C (n=45)	p-Value
Age (years), median (min-max)	66 (31-94)	68 (33-88)	0.5
Gender, n (%)			0.6
Male	61 (44)	21 (47)	
Female	79 (56)	24 (53)	
Primary cancer site, n (%)			0.3
Breast	24 (17)	6 (13)	
Urologic	17 (12)	2 (4)	
Gynecologic	13 (9)	3 (7)	
High digestive	30 (21)	11 (24)	
Colorectal and anal	26 (19)	11 (24)	
Pancreatic	7 (5)	4 (9)	
Liver and biliary tract	9 (6)	2 (4)	
Head and neck	7 (5)	2 (2)	
Melanoma	1 (1)	1 (2)	
Thoracic	2 (1)	1 (2)	
Unknown primary	4 (3)	2 (4)	
Cancer staging, n (%)			0.1
Locally advanced	5 (4)	0 (0)	
Metastatic	135 (96)	45 (100)	
Cancer treatment, n (%)			0.1
Polychemotherapy	22 (20)	4 (11)	
Monochemotherapy	26 (24)	7 (18)	
Hormone therapy	5 (5)	2 (5)	
Checkpoint inhibitors	2 (2)	-	
Tyrosine-kinase inhibitors	4 (4)	1 (3)	
Best supportive care	49 (45)	24 (63)	
Previous treatment lines (n), median (min-max)	2 (0-8)	2 (0-8)	-

The demographic and disease-related characteristics of patients from PaP Score Group C were similar to those of the overall population. The main difference was the higher percentage of patients under BSC at the time of admission in PaP Score Group C (63% vs 45%).

More than half of the patients (n=81, 57.9%) were hospitalized for symptomatic control (n=44, 31%) or disease progression with PS deterioration (n=37, 26%). Concerning causes of death, disease progression was presumed in 71% of cases. On the other hand, 28% of the patients were admitted because of an infectious event, and in 16%, it was considered that this clinical condition may have contributed to the patient’s death.

The data related to the IMs and PIMs prescribed at hospital admission and in the 24 hours prior to the patient's death are reported in Table 2.

**Table 2:** IMs and PIMs prescribed at hospital admission and in the 24 hours prior to the patient's death, in the overall population and according to PaP score. ^a^Except potassium, calcium, magnesium, and iron. ^b^Anti-hypertensive agents (except diuretics), antiarrhythmic drugs, and antiaggregants. ^c^H1 Blockers, proton pump inhibitors, antacids. PaP: palliative prognostic score; IMs: inappropriate medications; PIMs: potentially inappropriate medications.

Prescribed IMs and PIMs	At Hospital Admission				Last 24 Hours of Life			
	Overall population (n=140), n (%)	PaP score group			Overall population (n=140), n (%)	PaP score group		
		A (n=45), n (%)	B (n=50), n (%)	C (n=45), n (%)		A (n=45), n (%)	B (n=50), n (%)	C (n=45), n (%)
IMs	13 (9)	8 (6)	5 (4)	0 (0)	6 (4)	3 (2)	1 (1)	2 (1)
Statins	5 (4)	3 (2)	2 (1)	0 (0)	2 (1)	1 (1)	1 (1)	0 (0)
Anti-diabetic medication	1 (1)	0 (0)	1 (1)	0 (0)	0 (0)	0 (0)	0 (0)	0 (0)
Hormone replacement therapy	3 (2)	2 (1)	1 (1)	0 (0)	4 (3)	1 (1)	1 (1)	2 (1)
Vitamins and some minerals^a^	6 (4)	4 (2,9)	2 (1)	0 (0)	1 (1)	1 (1)	0 (0)	0 (0)
PIMs	119 (85)	42 (30)	38 (27)	38 (27)	100 (71)	37 (26)	37 (26)	26 (19)
Cancer treatment	9 (6)	6 (4)	1 (1)	2 (1)	2 (1)	2 (1)	0 (0)	0 (0)
Megestrol acetate	1 (1)	0 (0)	0 (0)	1 (1)	1 (1)	0 (0)	0 (0)	1 (1)
Cardiovascular drugs^b^	42 (30)	16 (11)	12 (9)	14 (10)	30 (21)	10 (7)	11 (8)	9 (6)
Anticoagulants	59 (42)	23 (16)	20 (14)	16 (11)	42 (30)	19 (14)	14 (10)	9 (6)
Gastric protectors^c^	96 (69)	33 (24)	30 (21)	33 (24)	83 (59)	30 (21)	31 (22)	22 (16)
Allopurinol	2 (1)	1 (1)	0 (0)	1 (1)	1 (1)	0 (0)	0 (0)	1 (1)

At the first day of hospitalization, 119 patients (85%) were exposed to at least one IM or PIM, mainly cardiovascular drugs (n=42, 30%), anticoagulants (n=59, 42%), and gastric protectors (n=96, 69%). There was a reduction in the number of IMs and PIMs in 19 of the 119 patients (16%), but 100 patients (71%) were still exposed to at least one IM or PIM in the last 24 hours of life, 88 patients (63%) had one to two PIMs prescribed and 12 patients (9%) had three to four PIMs.

According to the PaP score, all groups had PIMs prescribed at hospital admission; however, there was a therapeutic adjustment from admission to the last 24 hours of life, as described in Table 3.

**Table 3:** IMs and PIMs according to PaP score, at admission and at the last 24 hours prior to death. The statistically significant values are given in bold. PaP: palliative prognostic score; IMs: Inappropriate medications; PIMs: potentially inappropriate medications.

PaP Score P(IM)s	Group A (n=45)			Group B (n=50)			Group C (n=45)			Group C vs Non-Group C	
										p-Value	
Timing	At admission	Last 24h	p-value	At admission	Last 24h	p-Value	At admission	Last 24h	p-value	At admission	Last 24h
IMs, n(%)	8 (18)	3 (7)	0.04	5 (10)	1 (2)	<0.01	0 (0)	2 (4)	-	0.03	0.7
1-2 PIMs, n(%)	32 (67)	33 (73)	0.08	33 (66)	34 (68)	<0.01	31 (69)	21 (47)	<0.01	0.7	0.02
3-4 PIMs, n(%)	10 (22)	4 (9)		5 (10)	3 (6)		7 (16)	5 (11)			

Regarding PaP score group A, a reduction was evident in the prescription of IMs, with statistical significance (p=0.04), but the reduction in the prescription of PIMs was not statistically significant. In group B, the reduction occurred both in the prescription of IMs and PIMs, with statistical significance (p<0.01).

Regarding PaP score group C, which includes patients with a worse prognosis, there was no prescription of IMs at hospital admission. However, IMs were prescribed in two of the patients in the last 24 hours of life. Also, in this group, there was a significant reduction of PIMs (p<0.01) in the last 24 hours of life.

An analysis was made to determine whether there was a greater therapeutic adjustment in the group with the worse prognosis when compared to the other patients (group C vs non-group C). There was a lower prescription of IMs at admission with statistical significance (p=0.03) and, in the last 24 hours prior to death, this difference was also observed for the PIMs prescription (p=0.02).

A comparative analysis of PaP score groups A and C, regarding the reasons for hospitalization, showed in group C a higher proportion of hospitalizations due to uncontrolled symptoms (36% vs 24%) and disease progression (33% vs 20%), with no significant difference in hospitalizations due to infectious diseases (27% vs 29%).

Throughout hospital stay, a higher number of infectious events occurred in group A, diagnosed in 15 patients (33%) vs 4 (9%) in group C. As for other complications, namely those related to cancer disease (intestinal obstruction, malignant pleural effusion, hypercalcemia, pathological fracture, etc.), they occurred in 15 patients (33%) in group A vs 7 (16%) in group C; and other acute complications (pulmonary embolism, heart failure, atrial fibrillation, gastrointestinal bleeding, etc.) were identified in 9 patients (20%) in group A vs 3 (7%) in group C. Moreover, the length of hospital stay was higher in group A, with a median of 20.5 days (1-64), compared to group C, with a median of 6.5 days (0-28).

Therapeutic simplification was performed in 43% of the overall population. When analyzing the PaP score groups, therapeutic simplification was higher in group C, but not statistically significant (p=0.09; Table 4).

**Table 4:** Prevalence of therapeutic simplification in the overall population and according to PaP score. PaP: palliative prognostic score.

	Overall Population (n=140), n (%)	PaP Score Group		
		A (n=45), n (%)	B (n=50), n (%)	C (n=45), n (%)
Therapeutic simplification	50 (43%)	17 (38%)	13 (26%)	20 (44%)

Regarding ChT, 66 patients (47%) had ChT in the last two months of life, 38 (27%) in the last month, and 17 (12%) in the last two weeks prior to death. As for supportive therapy, most patients had symptomatic treatment prescribed at hospital admission (n=137, 98%). Table 5 shows the number of patients treated with the different classes of supportive care drugs. The main drug classes prescribed were opioids (n=108, 77%), anti-emetics (n=94, 67%), non-opioid analgesics (n=92, 66%), laxatives (n=73, 52%), and corticosteroids (n=73, 52%). During hospitalization, a review and adjustment of the prescribed supportive therapy occurred, mainly through an increase in the prescription of opioids (n=129, 92%, p<0,01) and antipsychotics/sedatives (n=96, 69%, p<0.01).

**Table 5:** Symptomatic treatment prescribed at hospital admission and at the last 24 hours of life, in the overall population (n=140).

Symptomatic Treatment	At Hospital Admission, n (%)	Last 24 Hours of Life, n (%)
Opioids	108 (77)	129 (92)
Non-opioid analgesics	92 (66)	85 (61)
Antidepressants	21 (15)	21 (15)
Antiepileptic drugs	11 (8)	11 (8)
Antipsychotics/sedatives	56 (40)	96 (69)
Benzodiazepines	62 (44)	71 (51)
Laxatives	85 (61)	89 (64)
Corticosteroids	73 (52)	82 (59)
Antiemetics	94 (67)	98 (70)

Accounting for potential predictors of therapeutic simplification and after univariate analysis, we produced a multivariate regression model to assess independent predictors of therapeutic simplification in EoL. The following variables were considered and analyzed: gender (female), age, hospitalization time, cause of hospitalization (infection), ChT free interval, performing ChT at hospitalization, stage IV disease, PaP score group C, inpatient consultation by the palliative care team, prescription of supportive therapies at admission, PIMs, and IMs at admission.

As shown in Table 6, PaP score group C (OR 2.44, 95% CI 1.03-5.80, p=0.04), inpatient consultation by the palliative care team (OR 3.86, 95% CI 1.22-12.2, p=0.02) and prescription of PIMs at admission (OR 2.33, 95% CI 1.37-3.97, p<0.01) were independent predictors of therapeutic simplification. 

**Table 6:** Univariate and multivariate logistic regression for predictors of therapeutic simplification in EoL. *Statistically significant. ChT: chemotherapy; PaP: palliative prognostic score; ICPCT: Inpatient Consultation by the Palliative Care Team; IMs: inappropriate medications; PIMs: potentially inappropriate medications.

Variables	Univariate Analysis			Multivariate Analysis		
	Odds ratio	95% CI	p-Value	Odds ratio	95% CI	p-Value
Gender (female)	1.4	0.68-2.97	0.4	-	-	-
Age	1.0	0.97-1.02	0.8	0.99	0.96-1.02	0.9
Hospitalization time	1.01	0.99-1.04	0.2	-	-	-
Cause of hospitalization (infection)	0.98	0.43-2.21	0.9	-	-	-
ChT free-interval	1.0	0.99-1.00	0.1	-	-	-
ChT at hospitalization (yes)				-	-	-
Cancer staging (IV)	0.74	0.45-1.92	0.8	-	-	-
PaP score (group C)	1.96	0.89-4.31	0.09*	2.44	1.03-5.80	0.04
ICPCT (yes)	2.87	0.98-8.38	0.05*	3.86	1.22-12.2	0.02
Supportive therapies at admission (yes)	1.17	0.91-1.49	0.2	-	-	-
PIMs at admission	2.08	1.27-3.42	0.004*	2.33	1.37-3.97	< 0.01
IMs at admission	1.28	0.48-3.40	0.6	-	-	-

## Discussion

There is an effort to achieve greater therapeutic suitability in palliative patients. However, many patients maintain futile and disproportionate therapy at the EoL. In many cases, systemic cancer treatment is performed until quite late in the course of the disease. Analyzing the group of patients who underwent ChT in the last month of life, there are some aspects that may justify these results, namely the fact that almost half of the patients belonged to the PaP score group A, that most patients underwent few lines of treatment, and that a significant percentage of patients had complications during hospitalization (infectious, cancer-related, or other acute clinical conditions), which may have led to the patient's death sooner than expected. For PaP score group C patients, there was a higher percentage of patients under BSC, when compared to the overall population, which may reflect greater therapeutic adequacy in patients with a worse prognosis.

The prescription of PIMs was significantly higher than that of IMs, which could be expected given their definition. Since IMs do not have a beneficial effect on symptomatic control, quality of life, or survival, their suspension is easier in patients with evidence of advanced cancer disease and uncontrolled symptoms. On the other hand, the decision to start or suspend PIMs is more complex, as their futility is more difficult to assume because multiple variables must be considered, such as their purpose, possible benefit, patient comorbidities, PS, and life expectancy. This complexity may explain the high prevalence of PIMs in the studied population, both at admission and in the last 24 hours of life.

For patients in the PaP score group A, there was a significant reduction in the prescription of IMs throughout the hospital stay, but not of PIMs. These were the patients with a higher life expectancy at hospital admission, who may still benefit partially from these drugs, which would explain the absence of significant therapeutic review and reduction.

Therapeutic simplification, as in suspension of all IMs and reduction of at least 50% of PIMs during hospitalization, occurred in less than half of the overall population (43%). Group C showed the highest rate of therapeutic simplification, but not statistically significant.

A predictor of therapeutic simplification in EoL was the patient's inclusion in PaP score group C. A shorter life expectancy at admission led to a greater therapeutic simplification. Intervention by the Palliative Care Team was also a predictor of therapeutic simplification, which can be explained by the more focused approach towards quality-of-life improvement and symptomatic control. Different than expected the prescription of supportive therapies at hospital admission was not a predictor of therapeutic simplification.

Limitations of this study include its retrospective design and, consequently, all limitations inherent to this condition. Being a single-center study is also a limitation. The collection of clinical data from medical records limits access to certain information. Another limitation is the prognostic score used. The CPS variable is subjective, and as such, prone to variations according to the clinician. An additional limitation is that two of the six criteria used in this score are often high in infectious diseases, which may overestimate the final score.

The maintenance of IMs and PIMs, as well as cancer treatments, until the last weeks or days of the patient's life is quite common, as already described in various published studies on the subject, and consistent with these results [10,11,12,14]. Although there was a reduction in IMs and PIMs in the studied population, and therapeutic simplification occurred in one fraction of the patients, the fact is that more than half of the patients evaluated did not undergo therapeutic simplification as defined in this work.

The future purpose of this work will be to develop a protocol to be applied in cancer patients, mainly in patients at the EoL, in order to assess therapeutic adequacy, with possible withdrawal of disproportionate therapies.

## Conclusions

It is important to understand the impact that the maintenance of unnecessary therapy has on the patient’s quality of life. The lack of benefit, the possibility of harmful drug interactions, the burden associated with taking many pills, must be considered in EoL patients. This can contribute to less therapeutic compliance, leading to uncontrolled symptoms and more (eventually preventable) hospital admissions.

It is of paramount importance to change the standard of care in EoL, and therapeutic review and simplification must be a goal in these patients. The recognition of flaws and the use of tools that help to address these patients and their needs are crucial to improve practice and care.
